# Assessing the Hybrid Effects of Neutral and Niche Processes on Gut Microbiome Influenced by HIV Infection

**DOI:** 10.3389/fmicb.2019.01467

**Published:** 2019-07-03

**Authors:** Guanshu Yin, Yao Xia

**Affiliations:** ^1^The Second Affiliated Hospital of Kunming Medical University, Kunming, China; ^2^Kunming Institute of Zoology, Chinese Academy of Sciences, Kunming, China; ^3^Kunming College of Life Science, University of Chinese Academy of Sciences, Kunming, China

**Keywords:** neutral theory, niche theory, microbiome analyses, hybrid model, HIV

## Abstract

That both stochastic neutral and deterministic niche forces are in effect in shaping the community assembly and diversity maintenance is becoming an increasingly important consensus. However, assessing the effects of disease on the balance between the two forces in the human microbiome has not been explored to the best of our knowledge. In this article, we applied a hybrid model to address this issue by analyzing the potential effect of HIV infection on the human gut microbiome and adopted a further step of multimodality testing to improve the interpretation of their model. Our study revealed that although niche process is the dominant force in shaping human gut microbial communities, niche process- and neutral process-driven taxa could coexist in the same microbiome, confirming the notion of their joint responsibility. However, we failed to detect the effect of HIV infection in changing the balance. This suggests that the rule governing community assembly and diversity maintenance may be changed by the disturbance from HIV infection-caused dysbiosis. Although we admit that the general question of disease effect on community assembly and diversity maintenance may still be an open question, our study presents the first piece of evidence to reject the significant influence of diseases.

## Introduction

Human gut is an ideal micro ecosystem colonized by countless microbes, where the mechanistic explanation of species abundance distribution (SAD) needs to be clarified. Typically, the forces that shape and maintain the biodiversity of community are thought to be controlled by deterministic factors, such as host species, genotype, diet, health, competition and niche differentiation, which has been referred to *Niche Theory*, but it fails to explain a number of rare taxa could coexist in very diverse environments when applied to macro-organisms (Ofiteru et al., 2010; Burns et al., 2015). The *Neutral Theory* proposed by Hubbell and widely used in the macro-ecology area nowadays has challenged this view. This theory considers trophically similar species are functionally equivalent and the SAD patterns in community can be explained by stochastic processes (Hubbell, 2001, 2006). The *Neutral Theory* combines neutrality, stochasticity, sampling and dispersal and presents a simple null model to test the mechanism of community assembling and biodiversity maintenance in ecological communities, but its abundance-based simplicity also arises query that is was thought by someone not robust enough, because many parameters may cause similar result (Alonso et al., 2006; Ofiteru et al., 2010; Li and Ma, 2016). Although contentious debates on both theories have been provoked, there have been adequate evidences supporting the idea that neither of them alone is sufficiently to explain the full range of observed SAD in natural communities (Stokes and Archer, 2010). The hypothesis that two contrasting theories are probably jointly responsible for the community assembly (Gravel et al., 2006; Leibold and McPeek, 2006; Zhang et al., 2009; Dumbrell et al., 2010; Stegen et al., 2012) seems more reasonable than either theory alone.

The ecological theories accounting for the community assembly have been tested in macro community widely until now, and their uses on microbial community have been gradually recognized recently, although there is still a long way to completely understand the microbial community. Schmidt et al. (2015) revealed that deterministic processes drive fish microbiome assembly dominantly while no evidence supports stochastic theory. O’Dwyer et al. (2015) also tested neutral theory for several datasets to determine whether or not the observed SAD patterns can fit the neutral prediction and found a clear departure from the predictions of standard neutral theories, indicating that standard neutral models may not provide the most useful null models for microbial communities. Li and Ma (2016) tested more than 7000 samples from different parts of human body with neutral theory, which revealed that very few microbial communities passed the neutral prediction. Given the hypothesis of hybrid effects of neutral and niche theory, it is too arbitrary to draw a conclusion that neutral processes do not play a role in microbial community. In this study, with the aim to identify the possible neutral process within non-neutral microbial communities, we used a hybrid model considering both neutral and niche theory to test a dataset of human gut sample. As the dataset we used contains samples from healthy individuals and patients with HIV that is proven to cause dramatic dysbiosis of gut microbiome (Lozupone et al., 2013; Mutlu et al., 2014; Dubourg et al., 2016; Williams et al., 2016), our another aim is to recognize the alteration of the mechanism of gut microbiome assembly resulted from HIV infection.

As mentioned before, it is difficult to identify quantitatively the exact processes shaping community from certain SAD patterns with standard neutral theories empirically. Quantifying the relative roles of neutral and niche process brings a non-trivial task. Microbial community is usually characterized by rich biodiversity, suggesting a high possibility that at least subpopulations controlled mainly by neutral process exist in a community. In a global view, such community consisting of both neutrally assembly and niche-selected taxa may not be recognized as a neutral community *via* neutral models concerning SAD pattern alone. Hence it is necessary to adopt more information, such as phylogenetic analysis, to build sophisticated models to evaluate the relative roles of both processes. Jeraldo et al. (2012) proposed a model that fuses measures of abundance with phylogenetic information that has been attracting increasingly attention in ecological studies (Kelly et al., 2008; Cavender-Bares et al., 2009; Jabot and Chave, 2009; Burbrink et al., 2015) to address this problem, which is particularly suitable for microbial community characterized by high level of biodiversity. The authors identified successfully subpopulations of the chicken gastrointestinal tracts that may be undergoing neutral process *via* the observation of a small non-zero peak within the distance-based plot generated by their genomic-based model. However, observations from histograms may sometimes be hard to determine and even misrepresent the real distributions. Therefore, we went a further step by adopting a multimodality statistical test, Silverman (1981), to improve the interpretation of Jeraldo et al. (2012) model.

## Materials and Methods

### Dataset Reprocessing

In Lozupone et al. (2013) study, they collected fecal samples of individuals with chronic HIV infection (*n* = 22), recent infection (*n* = 3), and HIV-negative controls (*n* = 13) on sterile swabs either during or 12 hr prior to the visit and then all individuals were assigned into four cohorts: (1) recent HIV-1 infection, individuals likely infected within the prior 6 months; (2) chronic HIV-1 infection untreated, individuals infected for >6 months and ART drug naive or off treatment for >6 months; (3) chronic HIV-1 infection on long-term ART, ART treatment for ≥12 months with a minimum of three ART drugs prior to study entry and viral suppressed for >6 months; and (4) healthy controls. Some individuals donated samples twice and 58 samples entered into the 16s rRNA sequencing process finally. The reads for each sample were stored at EBI^1^ (Accession Number ERP003611). In our study, we downloaded the sequencing data from EBI and selected qualified samples with enough numbers of high quality sequences to perform the recalculation.

We used Jeraldo et al. (2012) pipeline (Tornado^2^) to reanalyze the reads data. In brief, short sequences (shorter than 100 bp) and chimeras were eliminated firstly and remaining sequences were aligned using Mothur and Silva reference database. To make sure the sequences start and end at the same position, the ends of all alignments were trimmed. Then operational taxonomic units (OTUs) were picked with the complete linkage method of Schloss et al. (2009) with a cutoff of 3% sequence identity. All reprocessed OTUs entered the following analysis.

### The Computational Procedure for the Hybrid Model

For each sample, the observed OTUs can be classified into two categories: modal OTUs (most abundant) and rare OTUs (less abundant), on the basis of a threshold value *k*. The core idea is to visualize the correlations between modal and rare OTUs, which will depend on the ecological dynamics, using the information obtained from the phylogenetic distances of representative sequences between both types of OTUs and the abundances of OTUs in a hypothetic high-dimensional sequence space.

In the first case, suppose that a community is drove by neutral process, hence modal and rare OTUs in this community would distribute at random in the hypothetic space. The distances between OTUs (representative sequences) can be measured using a normalized Hamming distance:

(1) $$H_{i\,j}=\frac{1}{L}\,\sum_{\alpha =1}^{L}\left[1-\delta \,\left(S_{\alpha }^{i}-S_{\alpha }^{j}\right)\right]$$

where *H* is the distance between the *i*th and *j*th OTU, *L* is the length of the representative sequence (to compare the Hamming distance, all the analyzed sequences should be in the same length), δ is the Kronecker delta and *S* represents the label of base at a given position (from α to *L*) in the sequence with superscripts (e.g., *i* and *j*) indicating OTUs. *S* takes values 1, 2, 3, 4 corresponding to the four bases *ACGT*.

Ideally, in a neutral process-driven community, the mean of *H* should be 3/4 as the chance that two bases at the same position are identical is 1/4. However, considering the complications deriving from highly conserved bases that cannot be appropriately modeled as being chosen randomly from the alphabet in reality, the actual value of the mean of *H* would be *3(L-M)/4L*, given there are *M* conserved bases in the sequence. Then, for each rare OTU (labeled *k*), the distances between it and all modal OTUs are calculated *via* method described above and the shortest one is selected and labeled *E_*k*_*. {*E_*k*_*} is a subset of {*H_*ij*_*} and its distribution is also a bell-shape plot peaked at a slightly smaller value than mean {*H_*ij*_*}.

In another case, i.e., when the community is driven by niche process, the distributions of both types of OTUs are not random, where rare OTUs that evolve from modal OTUs through a few point mutations surround the modal OTUs closely. Such distributions are also observed in Jeraldo et al. (2012) study using a weighted version of principal component analysis (PCA) to reduce the hypothetic space into a 2D space, which are obviously different from the distributions in neutral community. All the normalized *H_*ij*_* from each rare OTU to the nearest modal OTU are calculated *via* making a Voronoi polyhedron construction in the hypothetic space. Thus, the probability distribution of *E_*k*_* should be a delta distribution that is peaked at *E* = 0 and decreases monotonically for *E* > 0.

In most cases, both neutral and niche process will not be responsible for the construction of the community solely therefore the hybrid effect should be took into account. Jeraldo et al. (2012) evaluated the hybrid effect of model when there are α*N* OTUs undergo a niche dynamic in a community containing *N* OTUs using Monte Carlo simulations on a simulated dataset. The parameter α can serve as an indicator suggesting observed community is driven by purely neutral process (α = 0), purely niche process (α = 1) or hybrid process (α is between 0 and 1). The distributions of distance plot in three types of community are clearly distinct in shape: for purely neutral community, the distribution plot is bell-shaped; for purely niche community, the distribution is a delta distribution that is peaked at *E* = 0 and then decreases for *E* > 0 (the authors found for niche-like models, the peak at zero moves to a non-zero peak that corresponds to the average size of the niche); for hybrid community, the distribution plot shows characteristic of niche distribution at the first-member (i.e., starts at a non-zero peak and then decreases monotonically) and characteristic of neutral distribution at the end-member (i.e., a non-zero peak arises in the end-member). The non-zero peak at the end-member can be viewed as an evidence for the presence of subpopulations shaped mainly by neutral process and the sequences within the peak may be undergoing neutral dynamics.

Jeraldo et al. (2012) also tried to improve their model by weighting the contribution of *E_*k*_* by abundance of OTU but failed to find change in the neutral community and qualitative change in the niche community, suggesting the distribution of distance may be only weakly dependent on the abundance distribution of OTUs. To simplify the calculation, the information of OTU abundance is not included in such analysis. Another concern is the choice of the threshold *k*. In the original study, the authors revealed that the results of the metric on model systems are unchanged when *k* is changing between 2 and 10% hence they select 1 to 10% for this parameter in their pipeline. In our study, we set the *k* value as 5%, which is used by the authors to present their experimental data in the original article. The complete computational procedure was performed in the softer ware Tornado see text footnote 2.

### Multimodality Test for the Distribution of Nearest Distances

The mode of a distribution is the value having highest probability of being observed. On the basis of the principle of their model, the non-zero peak at the right side of the histogram of nearest distance represents the neutral-driven subpopulations in a community. Thus, a pure niche process-driven community should have only one mode, and community driven by niche-neutral hybrid process should have more than one mode. Given the pure neutral community did not exist in the real world, the unimodality should represents niche process and multimodality should be equivalent to hybrid process. Silverman (1981) provides a classical method to test the null hypothesis that a distribution has at most *n* modes (*n* = *1* in our study), where the alternative hypothesis that the distribution has more than n modes can be rejected on the basis of a *p*-Value. In this study, for each sample, we draw both histogram and kernel density plot using the distribution of distance to the nearest modal OTU of every rare OTU in the sample community with *R* (version 3.3.2) and conducted Silverman (1981) with the *R* package, *silvermantest*^3^.

## Results and Discussion

**FIGURE 1:**
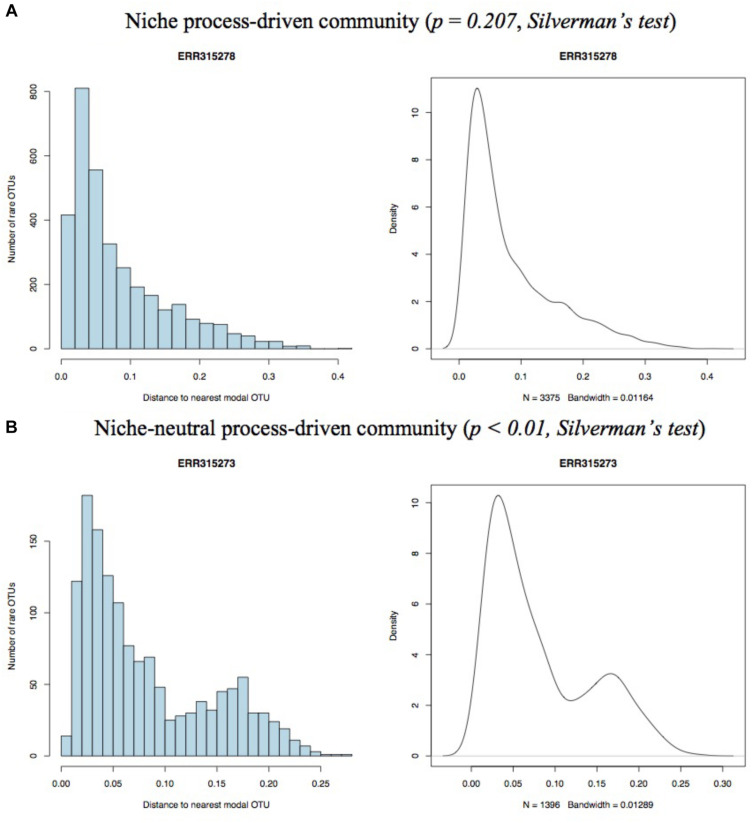
Representative histograms (left) and kernel density plots (right) for niche process- **(A)** and niche-neutral process-driven **(B)** community.

Only samples with enough numbers of high quality sequences were included in our study. In total, 52 qualified samples with 4 in cohort (1), 18 in cohort (2), 8 in cohort (3), and 21 in cohort (4) were selected. As the number of individuals in four cohorts are not balance, we here assigned cohort 1, 2, and 3 into HIV group and cohort 4 into Non-HIV group and compared the number of samples that fitted neutral-niche plot between two groups. For each sample, we generated both histogram and density plot on the distance of each rare OTU to nearest modal OTU and performed Silverman’s test for multimodality. Two pairs of representative histograms and density plots from niche process- and hybrid process-driven community, respectively, are displayed in Figure 1. The remains are shown in Supplementary Figure S1 and results of Silverman’s test are listed in Supplementary Table S1. The number of samples passing Silverman’s test for each cohort and group are displayed in Table 1. In detail, 56.86% (29/51) samples in total passed the Silverman’s test (*p* < 0.05), indicating they are undergoing niche-neutral process, thus 43.14% (22/51) samples are driven by niche process dominantly. According to different cohorts, 25% (1/4) samples in cohort (1), 72.22% (13/18) samples in cohort (2), 75% (6/8) samples in cohort (3) and 42.68% (9/21) samples in cohort (4) passed the multimodality test. No one sample satisfied the pure neutral plot, so the remains in each cohort are niche-driven community, suggesting that although niche process is the dominant force in shaping gut microbiome assembly and play roles in all samples, the contributions made by neutral process should not be neglected.

**TABLE 1:** The number of samples passing Silverman’s test.

**Group**	**Cohort**	**Samples driven by niche-neutral process**	**Samples driven by niche-neutral process**
HIV infection	Cohort 1	25% (1/4)	66.67% (20/30)
	Cohort 2	72.22% (13/18)	
	Cohort 3	75% (6/8)	
Non-HIV infection	Cohort 4	42.86% (9/21)	42.86% (9/21)

Because the number of individuals in four cohorts are not balance, we than assigned cohort 1, 2, and 3 into HIV group and cohort 4 into Non-HIV group and compared the number of samples that fitted neutral-niche plot between two groups. In HIV group, 66.67% (20/30) samples fitted neutral-niche plots and 42.86% (9/21) fitted neutral-niche plots in Non-HIV group. There is no significant difference between the results of two groups (*p* = 0.1504, *Fisher’s exact test*), indicating HIV infection may not change the force that shapes gut microbiome assembly essentially.

Four representative neutral-niche plots and niche-like plots for each cohort are displayed in Figure 1 and remaining plots can be found in Supplementary Figure S1.

It has been generally accepted that the community assembly is an important topic in the macro-community ecology area and can be accounted for by different ecological theories (Niu et al., 2009; Latombe et al., 2015). From the view of ecologists, however, the microbial community where the birth, death, immigration and speciation happen at any time is also an important subject that is controlled by general ecological principles and laws. Hence the two important theories explaining the assembly of community, niche theory underlying deterministic factors and neutral theory underlying stochastic factors, should be also appropriate to describe the microbial community assembly.

In this study, we performed a test of hybrid model considering both neutral and niche process on human gut samples and did not find one sample satisfy the prediction of pure neutral theory, which is consistent with our previous report, where we tested Human Microbiome Project (HMP) dataset with neutral models and found none of the gut samples passed the neutrality test (Li and Ma, 2016). The reason may be the high level of diversity and complexity of organisms colonizing in gut, just as the study by Fisher and Mehta (2014) suggests that the communities with large population sizes and relatively stable environment is more like driven mainly by niche-process and the communities with small population size and unstable environment is more likely driven by neutral-process. Fisher and Mehta (2014) also reveals the presence of a phase transition process between niche-driven phases and neutral-driven phases in communities, suggesting a neutral-niche phase where niche process and neutral process are jointly responsible for the assembly and maintenance of community should exist in amount of communities. Although our study confirmed that niche process play its role dominantly in shaping gut microbiome, our results also provides evidence that there are subpopulations driven mainly by neutral process in certain overall neutral communities, indicated by the observation of neutral-niche plots containing a small non-zero peak comparing with niche-like plots, where the sequences under the non-zero peak should be belong to the neutral-driven taxa. The coexist of neutral and niche process has been supported by many studies (Leibold and McPeek, 2006; Dumbrell et al., 2010; Ofiteru et al., 2010; Ayarza and Erijman, 2011), which may result from different physical reasons (Jeraldo et al., 2012), such as the fact that those generalist microbes that can exist in various environments constitute the neutrally assembly part of microbiome (Langenheder and Szeìkely, 2011), while the niche portion consists of those specific microbes that is adapted to the medium conditions (Burke et al., 2011). Disturbances of gut physiological environment may cause selections on microbes, especially for those niche process-driven taxa, hence we also wonder the question that whether or not the typical dysbiosis caused by HIV infection is linked to change of the force that shapes gut microbiome assembly. We compared the gut microbiome assembly forces between HIV infection and health and found that despite the different progress of HIV patients, the proportion of samples fitted niche-neutral plots in HIV group is 66.67% (20/30), which has no significant difference with the proportion of 42.86% (9/21) in health group, implying HIV infection would not change the rule that shapes gut microbiome assembly essentially.

Jeraldo et al. (2012) hybrid model provides a very effective tool to quantify the relative roles of niche and neutral processes shaping microbial community by merging measures of abundance with phylogenetic information, but there is room for improvement. First, it adopts distance-based histogram to measure the neutral-niche process, where the shape of plots sometimes is difficult to distinguish *via* observation, especially when the non-zero peak representing neutral process is not very evident. Second, the smoothness of distance plot is dependent largely on the number of OTUs, hence when the number of OTUs is not large enough, the shape of distance plot would be shapeless so that little information can be achieved. For the first issue, we adopted a statistical method, Silverman’s test, for testing multimodality *via* kernel density estimation, through which the niche-neutral hybrid process-driven community can be distinguished from niche community. As to the second issue, according to our experiences, for human microbiome samples, the gut samples usually can satisfy the requirement of the number of OTUs whereas other body parts such as lung and oral can hardly meet the number that generates eligible plots.

In summary, we firstly adopted a Silverman’s test on the original results of Jeraldo et al. (2012) hybrid model, which improved the interpretation of the results. Then we use this strategy to reanalyze a dataset of HIV-related human gut microbiome in order to find HIV-specific changes in the assembly of gut microbial community. Our results revealed that although niche process is dominant in shaping human gut microbiome, niche process- and neutral process-driven taxa could coexist in the same microbiome, confirming the idea that niche and neutral processes may be jointly responsible for the gut microbial community assembly and HIV infection-caused dysbiosis may not change the force that shape the assembly of gut microbiome. Besides the evidences that niche and neutral process may co-occurrence in gut microbiome, our study also offer suggestions for improvement of Jeraldo et al. (2012) model *via* introducing statistical multimodality test method.

Our study is a pilot study reanalyzing only one dataset with limited samples. This is the major limitation of our study. As there has not been a consistent HIV-specific dysbiosis pattern of gut microbiome, which reflects from the contradictory results of studies using different samples and datasets (Noguera-Julian et al., 2016). This may because gut microbiome is related to plenty of factors in addition to HIV infection, such as socioeconomic factors, geography, age, diet, drug use, genetic, lifestyle and sex preference (Noguera-Julian et al., 2016; Liu et al., 2017; Williams, 2019). For example, Noguera-Julian et al. (2016) found that the gut microbiome of men who has sex with men (MSM) was richer and more diverse than that of non-MSM men. Given the limited samples available for each study, it is challenging to control all the confounders in HIV-related gut microbiome study. Thus few studies have successfully established causal links between changes of the gut microbial community composition and HIV infection. Likewise, to investigate the HIV-specific change of assembly of gut microbial community is also a non-trivial task. In further study, we would try to collect more samples from different datasets and use a hierarchical analysis strategy to achieve more reliable results.

## Data Availability

The dataset analyzed for this study can be found in the EBI (http://www.ebi.ac.uk/ena/; Accession Number ERP003611).

## Author Contributions

YX conceived and designed the experiments, prepared the figures and tables, and drafted the manuscript. YX and GY carried out the experiments, analyzed the data, interpreted the results, and approved the final draft of the manuscript. GY reviewed the draft of the manuscript.

## Conflict of Interest Statement

The authors declare that the research was conducted in the absence of any commercial or financial relationships that could be construed as a potential conflict of interest.

## Footnotes

1 http://www.ebi.ac.uk/ena/

2 http://tornado.igb.uiuc.edu/bio/

3 https://www.mathematik.uni-marburg.de/∼stochastik/R_packages/
