# Corrigendum: Assessing the Hybrid Effects of Neutral and Niche Processes on Gut Microbiome Influenced by HIV Infection

**DOI:** 10.3389/fmicb.2021.710865

**Published:** 2021-06-09

**Authors:** Guanshu Yin, Yao Xia

**Affiliations:** ^1^The Second Affiliated Hospital of Kunming Medical University, Kunming, China; ^2^Kunming Institute of Zoology, Chinese Academy of Sciences, Kunming, China; ^3^Kunming College of Life Science, University of Chinese Academy of Sciences, Kunming, China

**Keywords:** neutral theory, niche theory, microbiome analyses, hybrid model, HIV

In the published article, there was an error regarding the affiliations for Yao Xia. As well as affiliation 2, he should also have a third affiliation of: *Kunming College of Life Science, University of Chinese Academy of Sciences, Kunming, China*.

In addition, equal contribution was not attributed to the authors. The statement “These authors have contributed equally to this work and share first authorship” should be added.

The authors apologize for this error and state that this does not change the scientific conclusions of the article in any way. The original article has been updated.

