# Supplementary material for: Assessing the Hybrid Effects of Neutral and Niche Processes on Gut Microbiome Influenced by HIV Infection
Source: Front Microbiol. 2019 Jul 3;10:1467. doi: 10.3389/fmicb.2019.01467 (PMC6639661; doi:10.3389/fmicb.2019.01467)
Supplement: Supplementary file 2 [file Table_1.DOC]

1

Table S1. Results of Silverman’s test

| **ID** | **Cohort** | **P-value** | **Process** |
| --- | --- | --- | --- |
| ERR315268 | 4 | 0.611 | Niche |
| ERR315269 | 1 | 0.717 | Niche |
| ERR315270 | 4 | <0.01 | Niche-neutral |
| ERR315271 | 2 | 0.767 | Niche |
| ERR315273 | 3 | <0.01 | Niche-neutral |
| ERR315275 | 2 | <0.01 | Niche-neutral |
| ERR315276 | 4 | <0.01 | Niche-neutral |
| ERR315277 | 4 | 0.898 | Niche |
| ERR315278 | 2 | 0.207 | Niche |
| ERR315279 | 2 | <0.01 | Niche-neutral |
| ERR315280 | 2 | <0.01 | Niche-neutral |
| ERR315281 | 3 | <0.01 | Niche-neutral |
| ERR315282 | 1 | 0.62 | Niche |
| ERR315283 | 2 | <0.01 | Niche-neutral |
| ERR315284 | 3 | 0.116 | Niche |
| ERR315285 | 4 | 0.318 | Niche |
| ERR315286 | 3 | <0.01 | Niche-neutral |
| ERR315287 | 2 | 0.063 | Niche |
| ERR315288 | 4 | 0.553 | Niche |
| ERR315289 | 4 | <0.01 | Niche-neutral |
| ERR315290 | 1 | 0.462 | Niche |
| ERR315291 | 3 | <0.01 | Niche-neutral |
| ERR315292 | 2 | 0.016 | Niche-neutral |
| ERR315293 | 4 | 0.572 | Niche |
| ERR315294 | 3 | 0.025 | Niche-neutral |
| ERR315295 | 2 | <0.01 | Niche-neutral |
| ERR315297 | 4 | 0.276 | Niche |
| ERR315298 | 1 | <0.01 | Niche-neutral |
| ERR315299 | 4 | 0.305 | Niche |
| ERR315300 | 2 | 0.14 | Niche |
| ERR315301 | 2 | <0.01 | Niche-neutral |
| ERR315302 | 3 | 0.088 | Niche |
| ERR315304 | 4 | 0.571 | Niche |
| ERR315305 | 4 | <0.01 | Niche-neutral |
| ERR315307 | 2 | <0.01 | Niche-neutral |
| ERR315308 | 4 | <0.01 | Niche-neutral |
| ERR315309 | 2 | <0.01 | Niche-neutral |
| ERR315310 | 2 | <0.01 | Niche-neutral |
| ERR315311 | 4 | <0.01 | Niche-neutral |
| ERR315313 | 2 | <0.01 | Niche-neutral |
| ERR315314 | 2 | 0.016 | Niche-neutral |
| ERR315315 | 4 | <0.01 | Niche-neutral |
| ERR315316 | 4 | <0.01 | Niche-neutral |
| ERR315317 | 4 | 0.255 | Niche |
| ERR315318 | 4 | 0.28 | Niche |
| ERR315319 | 4 | <0.01 | Niche-neutral |
| ERR315320 | 2 | 0.049 | Niche-neutral |
| ERR315321 | 4 | 0.239 | Niche |
| ERR315322 | 2 | 0.609 | Niche |
| ERR315323 | 4 | 0.788 | Niche |
| ERR315324 | 3 | <0.01 | Niche-neutral |
